# Too Black for Care: Clinical Apperception, Anti-Blackness, and Narrative Aporia

**DOI:** 10.1007/s11013-026-09984-1

**Published:** 2026-04-10

**Authors:** Roy Cherian

**Affiliations:** Humanities Gateway, University of California, 4100 Humanities Gateway, Irvine, CA 92697-3375 USA

**Keywords:** Clinical subjectivation, Anti-Blackness, Abstraction, Narrative medicine, Secularism

## Abstract

In this essay, I read Seth Holmes and Maya Ponte’s work on “en-casement” alongside Immanuel Kant’s *Critique of Pure Reason* to argue that faculties of biomedical apperception cultivated through clinical training are symptomatic of an orientation to sensational experience developed within Enlightenment philosophy. Characterized by the negation of subjectivity, en-casement is an expression of the anti-Black tendency to dehumanize and dominate the other in ways that render the biomedical paradigm of healing impotent with regard to the redress of Black suffering. I problematize narrative medicine as an intervention to resist en-casement by drawing on Afropessimism to elaborate limits and failures derivative from its assumptive logic of a free, agentive, autonomous, and sovereign subject capable of dramatizing suffering. Insofar as paradigmatic social death renders Blackness as a site of absolute dereliction on the level of the Symbolic, Black suffering is aporetic to narrative in ways that make even the humanist intervention no less impotent as a mode of redress. Given that the totalizing violence of anti-Blackness forecloses the redress of Black suffering within humanist paradigms of healing writ large, from the biomedical to the narrative, I consider the unmet demand for ante-anti-Black forms of care from the framework of abolition medicine.

## Introduction

At the heart of my argument is the assertion that Black emplotment is a catastrophe for narrative at a metalevel rather than a crisis or aporia within a particular narrative. To put it differently, social death is aporetic with respect to narrative writ large (and, by extension, to redemption writ large). (2020, pp. 226–227)

--Frank B. Wilderson, III

In this essay, I approach the redress of suffering within the biomedical paradigm of healing through Black studies to argue that the problems with biomedicine go beyond the dehumanizing processes of clinical diagnosis elaborated by medical anthropologists and humanists (Adams & Kaufman, 2011; Conrad, 2007; Fanon, 1959; Holmes & Ponte, 2011). I will argue that the problem with biomedicine sits at the very core of how suffering is symbolized and pain understood within the secular-republican tradition (Asad, 2011). To make this case, I will argue that the allopathic approach to the general redress of suffering through distillation, abstraction, and ultimately dehumanization within the biomedical paradigm of healing is symptomatic of Enlightenment philosophy undergirding secular-republican onto-epistemology. Further, drawing on Afropessimism, I will argue that the intervention of narrative medicine aimed at mitigating the dehumanizing effects of diagnosis and treatment within the biomedical paradigm is limited with regard to the redress of Black suffering. This limit, if not failure, of the humanistic turn in biomedicine demands alternative and ante-anti-Black^1^ (Sexton, 2012; Sharpe, 2012) paradigms to approach the problem of suffering, which will be considered through the framework of abolition medicine.

Put differently, I intend, following Michel Foucault, to take a critical attitude toward biomedicine in order to open an examination into the contingency therein with regard to “the preexisting relation linking will, authority, and the use of reason” (1984, p. 33) governing this particular and peculiar paradigm of healing. I excavate how with regard to the biomedical paradigm of healing what is “given to us as universal, necessary, obligatory… is singular, contingent, and the product of arbitrary constraints.” (Foucault, 1984, p. 43) By taking a critical attitude toward biomedicine, I throw into relief how the biomedical paradigm of healing and worldmaking “does not readily admit of the constraints by which that ordering takes place.” By arguing that, far from universal, biomedicine is a paradigm of healing “historically determined, to a certain extent, by the Enlightenment” (Foucault, 1984, p. 41), I throw into crisis “our most sure ways of knowing” the body in health and illness, in life and in death (Butler, 2002, pp. 225, 217). Rather than an exercise in judgment, I attempt to traverse the tense terrain of constrained generality in an interrogation of the clinical encounter that seeks to expose contingency while forfeiting any claims of, or prescriptive gestures toward, a new universality. Ultimately, the positivist and universalist assumptions of biomedicine’s allopathic approach to suffering crowds out alternative modes and manners of redress. Within biomedicine’s allopathic approach to suffering, those violently enacted as socially dead flesh, as non-beings without the proprietary relation to the body that comes with secular-republican forms of personhood, freedom, autonomy, and ultimately humanity are simply too black for care.

Furthermore, insofar as biomedical knowledge and practice are essential to modern modes of (de)subjection and subjugation, such an interrogation engages in a critical tradition invested in the radical destabilization of the forms of governance that characterize the modern secular-republican State, from the anatomo-political discipline of bodies to the biopolitical regulation of populations. In other words, it is a way out of being governed, by *them*, in *this* way. This interrogation grasps for an, exit, or an opening, toward radically new ways of thinking about life and death, health and illness, and the redress of suffering. In a word, it seeks a new way of caring. A way that does not, as I will argue, rely on anti-Blackness, on the negation of subjectivity and disavowal of suffering characteristic of the secular-republican onto-epistemological tradition inaugurated by Enlightenment philosophy and enshrined in the work of Immanuel Kant.^2^ If the problem of anti-Blackness at the core is a problem of ontology (Wilderson III, 2010), then for our purposes it is with the question of being, or more precisely being sick, that we must begin.

## Medical Ontology, Disease, and the Patient-Body

As skillfully argued in Annmarie Mol’s *The Body Multiple* (Mol, 2003) objects of medical ontology and epistemology, namely disease and the patient-body, are the products of enactment. Enactment is distinct from social construction, which assumes no opposition in the making of reality, as well as performance, which assumes a sort of staging of reality from a predetermined script. The concept of enactment emphasizes the contingency and contestation of medical ontology. Focusing on the enactment of disease and the patient-body through contestational discourse and practice allows for dynamic and multiple ways of attending to, interacting with, and shaping the patient-body to emerge. Rather than opposing the notion of medicine or its object (i.e., the body) as unified by suggesting fragmentation, Mol argues instead that “objects come into being—and disappear—with the practices in which they are manipulated. And since the object of manipulation tends to differ from one practice to another, reality multiples” (Mol, 2003, p. 5). As Mol demonstrates, the differentiation between actors, practices, and implements of the various enactments of disease creates multiplicity in the patient-body but not fragmentation insofar as these enactments are distributed across various, related sites of the hospital. This distribution allows for the coordination of these enactments to produce a diagnosis, inscribed within the clinical note (Mol, 2003).^3^ Medical ontology, or the enactment of disease and the patient-body, is not given but emerges through socio-material practices, from the dialogic clinical encounter to the nondialogic in the form of labs and imaging, that bridge multiple sites and involve numerous actors. This multiplicity is synthesized to render a diagnosis and treatment plan through processes of coordination and distribution across different sites of the hospital that come together in the clinical note.^4^

While clinical notes may now be accessible to patients, the power of inscription remains with medical professionals. In fact, it is in these processes of coordination that the exercise of power and clinical authority become most decisive and reigns supreme. The clinical encounter is only the beginning of this process and arguably the only, albeit recurring, stage in which the person plays an active role in diagnosis. Not soon after asking some variation of “where does it hurt,” the process of coordination leans toward allegedly more objective evidence through diagnostic technologies and treatment apparati only available to medical institutions and professionals, further narrowing the opportunity for contestation to occur. Clinical authority subtended by biomedical styles of reasoning and apparently objective modes of apprehending suffering gives certain representations greater influence in the enactment of disease and the patient-body. The illness narrative is only one of the many representations of suffering. Understood as subjective, they exist in the clinical note only in their distilled form, subordinated to more objective representations—from vitals to pathology labs and imaging. By creating distance between the object (i.e., disease) and the observed (i.e., the patient) as well as enhancing medical professional’s capacity for perception through advanced diagnostic and imaging technologies, these resources amplify claims to objectivity while negating subjectivity of both the clinician and the patient (i.e., “it is not I [who says this], it is the machine”) (Daston & Galison, 2010). In the end, biomedical forms of apprehending, understanding, and ultimately knowing suffering take precedence over patient presentations in the process of coordination, diagnosis, and treatment.

In the next section I will argue that this hierarchization of evidence and the process of diagnosis is governed by the secular-republican onto-epistemic tradition. By reading Seth Holmes and Maya Ponte’s case study on clinical subjectivation through en-casement alongside Kant’s *Critique of Pure Reason* (1781), I will argue that the enactment of disease and the patient-body within the biomedical paradigm of healing is symptomatic of the Enlightenment.

## En-casement and the Enlightenment: Clinical Apperception, Disease, and Suffering-in-Itself

However contested or multiple, the singular enactment of disease and the patient-body required to initiate biomedical treatment pathways categorically begins with the distillation of the phenomenal lived experience of pain and suffering represented in illness narratives into symptoms and problem lists in the clinical note, a process Seth Holmes and Maya Ponte call en-casement (Holmes & Ponte, 2011). As described by Holmes and Ponte, medical students are disciplined and habituated in ways that cultivate the critical faculty for clinical en-casement, a form of medical perception and style of reasoning that makes the often messy lived experience of suffering expressed in illness narratives digestible for biomedical consumption, distribution, and exchange through processes of distillation, abstraction, and clinical validation. To quote Holmes and Ponte at length,

medical students learn to replace the patient’s detailed and sometimes messy descriptions of their experiences with biomedical terminology that is easily and quickly understood by other members of the medical team…This has the effect of distancing the description of events from the personal human realm of experience (c.f. Cicourel, 1983) and categorizing the narrative into recognizable symptoms and signs for diagnostic and clinical consumption….This atomization of the illness narrative into actionable problems is necessary not only for the functioning of the biomedical system, but also for the production of medical student subjectivity (2011, pp. 177–178).

Through discourses and practices of en-casement, medical students are subjectivated and disciplined as clinicians and people into patient-bodies. Put differently, en-casement at once desubjectivates the person as a patient and subjectivates the medical student into a clinician. To the person being desubjectivated as a patient, the biomedically subjectivated ask—”Where does it hurt?” Such a question betrays the search for an abstract and depersonalized cause rather than a complex social apprehension of suffering. Through en-casement, both patient and clinician emerge as distinct subject positions as well as disease as an object of clinical intervention. Furthermore, in the process of enacting disease as object of clinical intervention through en-casement, critical distance is created between the object and the observed. Within the biomedical paradigm of healing this desubjectifying process of translation and representation is the necessary point of departure to catalyze the treatment pathway from an illness narrative. Over time, the cultivated capacity for en-casement diminishes the clinician’s capacity to hear, let alone appreciate, illness narratives, which become noise subordinated to validation by pathology labs and imaging. For the veteran clinician, enunciation and translation occur simultaneously. Resonating with Mol’s ethnography, the multiplicity of processes whereby socially embedded representations of illness are transformed, or translated, into clinical representations of disease, people into patient-bodies, and medical students into clinicians, converges in the disciplinary schema of the clinical note (Holmes & Ponte, 2011). The clinical note effectively logs the results of the “reductionist experiment of dissecting the patient’s narrative into…a problem-oriented patient presentation.” 5 Suffering is thus made amenable to biomedical modes of redress across various sites and through numerous and different actors. However effective in alleviating symptoms and prolonging life, such modes of redress come at the price of flattening the social relationship between pain, suffering, and lived experience for patient and provider alike.

The processes of en-casement whereby clinicians distill illness narratives into discrete list of symptoms indicative of clinical problems which they synthesize to enact a diagnosis and a patient-body derives from a particular, and perhaps even peculiar, style of reasoning. More precisely, given that the biomedical style of reasoning and mode of clinical understanding is characterized by the distillation of illness narratives into discrete lists of problems amenable for biomedical consumption, distribution, exchange, and ultimately intervention, I contend it is a paradigm of healing firmly rooted in the secular-republican onto-epistemological tradition, most comprehensively elaborated by Immanuel Kant. More directly, the biomedical paradigm of healing is symptomatic of Kantian thought in its movement from the phenomenality, the sensationality, and the particularity of illness of into the abstract, cognitive, and absolute of disease. In other words, the marked tendency toward the negation of subjectivity in illness narratives for the enactment disease and the patient-body in the biomedical paradigm of healing is “determined, to a certain extent, by the Enlightenment” (Foucault).

To interrogate this possibility, I ask a number of questions. First, while sensation is distinct from experience, which is distinct from cognition, is the inclination in biomedical paradigms of healing to rely on an abstract representation of suffering (e.g., vital signs, pathology labs, imaging technology), as opposed to illness narratives synthesized from empirical sensations intuited as suffering, symptomatic of a Kantian desire to excavate from the particularity of experience apprehended by the sensible intuition the absolute object of suffering-in-itself: disease? In other words, can we understand the movement from socially embedded understandings of illness toward biomedically abstracted disease by way of clinical understandings governed by processes of en-casement as an attempt to get at the objects-in-themselves of human suffering?

In the *Critique of Pure Reason* (1781), Immanuel Kant postulates the possibility for the synthesis of objective judgments despite the inherent spatial and temporal limitations of human perception. Arguably, these limits are impositions of secular materiality and metaphysics governed by the principle of finitude. Through a calculated movement toward progressive unity, for Kant the exercise of reason begins with the particularity of experience and arrives at the absolute of judgements. To break this down further, the intuition first organizes sensations of particular objects experienced in space and time into appearances, which are synthesized through the universal concepts of the understanding to engender cognitions (Kant, 1999, p. B151). These cognitions, or thoughts, are the raw material through which the dialectical exercise of transcendent and regulative reason form absolute ideas, or judgements (Kant, 1999). Although for Kant “objects in themselves are not known to us at all” (Kant, 1999, p. B45), the sensible intuition remains the beginning of all knowledge (Kant, 1999, p. B34).^6^ While for Kant human reason is limited to dealing with mere appearances, he nevertheless confers a level of objectivity upon the capacity for judgment by deducing self-consciousness, what he calls the unity of apperception, as the shared and universal condition of possibility for all possible intuition, and thereby shared cognition, among human beings.^7^ In other words, it is shared cognitive structure that allows for the totality of all possible sensible experience to appear, which is to say be intuited in space and time, to and by all human beings in the same way. The claim that perception of the sensible world is universal relies on the assumption of a shared structure (Merleau-Ponty & Carman, 2013), or an anatomical conception of the body. The shared (neuro)anatomical structure among human beings serves as an objective basis for an onto-epistemology that go on to inform secular-republican subjectivation and governance, clinical and otherwise. Thus, for Kant it is the unity of apperception that resolves the problem that all human beings truly have access to is appearances and not objects-in-themselves.^8^ In biomedical terminology, as the primary object of clinical judgement, suffering-in-itself is never known to the sufferer (i.e., the patient). Suffering-in-itself is even less accessible to the other (i.e., the clinician); nevertheless, it can be intuited from symptoms, conceptualized as illness, understood and synthesized as clinical problems, and ultimately judged as disease through the exercise of reason and the use of clinical technology, from pathology labs to advanced imaging apparatuses.

Cultivating the capacity for en-casement is integral to what I am calling the unity of clinical apperception. Just as the general unity of apperception makes possible objective judgements through the exercise of reason, so too does the unity of clinical apperception subtend the exercise of biomedical reason, which operates by distilling and abstracting illness narratives into discrete symptoms. Within the biomedical paradigm of healing, the person in pain is treated as any other object of sensational experience, as a mere appearance; the truth of their suffering can only be known through cognitive abstraction. The fact that these distilled symptoms are labeled in medical charts as “indications” gestures toward how these are mere appearances, external manifestations of the object of suffering-in-itself: disease. The exercise of biomedical reason then relates these symptoms, or indications, into physiological and metabolic problems, validated through objective technologies of calculation and enhanced perception housed in pathology and imaging labs which it synthesizes into a diagnosis and treatment plan. To make these generalized judgements, or diagnoses, biomedical reasoning operates on the assumption of a shared, standard, and universal human body just as Kant bases his unity of apperception on the shared anatomical structure of the brain. While sensation may be particular, or subjective, the spatial and temporal conditions of possibility in which all human being can experience illness and suffering (i.e., the body) allows for objective synthesis of general sensations of suffering as symptomatic of a particular disease. Given that all sensations remain bound within the temporal and spatial conditions of the human body, it is not the spatiality or temporality that biomedical paradigms of healing put in question. Rather what makes most illness narratives unsuitable for biomedical consumption, diagnosis, and ultimately intervention is its temporal and spatial *particularity*; in a word, what is problematic is its *subjectivity*. However, once unpleasant sensations (i.e., suffering) synthesized through narrative as illness become translated into clinical language as universal symptoms, they can become objectively related to each other in ways that signal something even more abstract: disease, or the absolute manifestation of suffering, which is to say suffering-in-itself. Given how the singularity of suffering frustrates representation to and apprehension by an other (Scarry, 1987), clinical en-casement and validation through labs and imaging become necessary for objective judgements, or clinicial diagnosis.

Insofar as biomedical diagnosis and intervention moves from the particular, sensational experience of suffering represented in illness narratives into absolute representations of disease through the intermediary deduction and synthesis of “complaints” into universal indications, symptoms, or problems, it is firmly embedded in secular-republican onto-epistemological tradition. To put it in Kantian terminology, if a symptom is the conceptual organization of sensations that cause suffering, disease is the synthesized judgement of suffering-in-itself. Governed by the unity of clinical apperception located in the anatomical structure of the body, the biomedical style of reasoning distills, translates, and re-presents a particular, sensational, embodied, and subjective experience of suffering represented in illness narratives into symptoms, which it ultimately synthesizes into an absolute, disembodied, and localized object of intervention amenable to clinical intervention called a “disease.” Arguably, the process of en-casement not only mirrors the calculated movement from the particular, to the universal, and finally the absolute in Kant, but is symptomatic of the same desire to suppress, if not negate, the subjectivity of suffering to confer objectivity upon clinical judgments.^9^ With this in mind, if biomedicine is accused of being dehumanizing, perhaps such accusations derive in no small part from its elaboration  within an onto-epistemological tradition forged in the Enlightenment that relies on the negation of subjectivity (i.e., personhood)—the self and the other—for the production of knowledge.

Despite the centrality of this (de)humanizing “reductionist experiment” of en-casement described by Holmes and Ponte (2011), narrative remains essential to initiating the biomedical treatment pathway, even if ultimately distilled and discarded. In this regard, illness narratives exist in peculiar tension with clinical apperception. On the one hand, illness narratives are the point of departure for the work of clinical apperception, understanding, and judgement—which is to say diagnosis and treatment. On the other, clinical apperception remains suspicious of patient presentations that cannot be validated through objective criteria, such as labs and imaging, and thus amenable to inscription in the clinical note. Pain is foremost among the symptoms notoriously intractable to objective measurement and en-casement, making it an object of suspicion, particularly when the pain is represented, or narrativized, by Black people.

## Narrative Aporia and Black Suffering

Given the marked tendency to desubjectivate people in pain as patient-bodies within the biomedical paradigm of healing, a tendency which we can now appreciate is born from a desire to get at the object of suffering-in-itself symptomatic of a particular orientation to knowledge cultivated in the Enlightenment, social scientists and humanists have put forth interventions aimed at mitigating the dehumanization associated with clinical en-casement. The critical role illness narratives play in initiating the treatment pathway is precisely why they have become a salient site for intervention and resistance, if not refusal, of processes of en-casement through the framework of narrative medicine. In answering the question “where does it hurt,” illness narratives, however abstracted, distilled, and discarded, are required to initiate the treatment pathway. Thus, even if illness narratives only remain in their distilled elements within the clinical note, they nevertheless remain critical in the initiating the treatment pathway within biomedical paradigms of healing. Thus, in order to enact disease and a patient-body in the first place, one must first be capable of representing through narrative to personal experience of suffering as general illness. However, the capacity to narrativize suffering and initiate treatment, even if desubjectivated and dehumanizing, is not the same for all those seeking care. While subjectivity and personhood are being valorized and reintroduced into the biomedical paradigm of healing through interventions informed by narrative medicine, to paraphrase Judith Butler, these interventions remains uncritical of the not readily admitted constraints surrounding narrative medicine’s (re)ordering of the clinical encounter. As I will argue, insofar as an exit in the sense described by Foucault is necessarily radically new, it is not enough to simply reinsert subjectivity into the clinical encounter without first critically examining the conditions of possibility for such prescriptive gestures and interventions.

Ostensibly, the capacity to narrativize suffering and enact illness is narrative medicine’s condition of possibility. The condition of its realization is the legibility of illness narratives, which allows for their recognition as evidentiary, reciprocity in the form of diagnosis, and incorporation in the apparati of biomedical forms of care through treatment. We have already considered how clinical subjectivation through en-casement troubles the legibility of illness narratives. To address this limitation, there are pedagogical efforts focused on integrating cultural humility and structural competency into clinical training (Metzl & Hansen, 2014; Yeager & Bauer-Wu, 2013). However, what if the first condition cannot be met? If one lacks the capacity to narrativize suffering, this particular avenue for redress is foreclosed. If one lacks this capacity, then the question becomes, what can biomedicine, an already desubjectifying and dehumanizing approach to the redress of suffering, do for those whose suffering does not only fall within the conventional structure of illness narratives but narrative writ large? The mutual constitution of patient and clinician through en-casement assumes that prior to processes of clinical (de)subjectivation, what we are dealing with are human beings. What happens when the being that suffers is not, to be precise, a being at all, let alone a human being? While well-intentioned, narrative medicine fails to redress the suffering of one whose capacity for narrative is foreclosed by social death, who is not a subject of rights but rather “an object in the midst of other objects” (Fanon, 1952).

Drawing on Frank Wilderson’s discussion of Black narrative aporia (Wilderson III, 2015, 2020), I want to suggest that paradigmatic social death forecloses the capacity to narrativize Black suffering, and thus engage in the intervention of narrative medicine. As Wilderson writes in *Afropessimism*,

At the heart of my argument is the assertion that Black emplotment is a catastrophe for narrative at a metalevel rather than a crisis or aporia within a particular narrative. To put it differently, social death is aporetic with respect to narrative writ large (and, by extension, to redemption writ large). (2020, pp. 226–227)

The constituent elements of social death as elaborated by Orlando Patterson (Patterson, 1982) work together to enact the Black “body” as always already diseased-flesh. Natal alienation obliterates the notion of a past prior to enslavement and symptomaticity. There is no space and time—past, present, or future—where Blackness is anything but pathological—symbolically, materially, or metaphysically. Gratuitous violence makes a material reality of the anti-Black fantasy that Blackness is deathliness incarnate (Marriott, 2016; Sexton, 2015). The consequent dereliction^10^ and ruination of Black life (Campbell, 2016) secures a state of general dishonor absolutely. Constituted by gratuitous violence, general dishonor, and above all natal alienation (Patterson, 1982), social death obliterates the moment of prior plenitude from which to narrativize suffering. Blackness is enacted as pathological and vertiginous, structured by violence that knows neither temporal nor spatial limitations. Further, this violence is read socially as the flat line of anti-Blackness,^11^ which renders Black suffering inarticulable on the level of grammar, which precedes and exceeds the level of articulation.

If narrative follows the general structure that arcs from equilibrium to disequilibrium to equilibrium restored, then it follows that an illness narrative takes the pattern health-illness-health restored. The enactment of disease and the patient-body in the clinic (2003) relies on representations of suffering and illness, as a deviation from a prior asymptomatic state through narrative. However, insofar as Blackness is materially, symbolically, and metaphysically positioned as pathological, as symptomaticity as such (Radhakrishnan, 2019), there is only disequilibrium without the possibility for equilibrium—restored or otherwise. The narrative arc of the socially dead is “a flat line that moves from disequilibrium, to a moment in the narrative of faux-equilibrium, to disequilibrium restored and/or rearticulated” (Wilderson III, 2020, 102). When faced with Black suffering, the faculty of clinical apperception can only see symptomaticity as the state of paradigmatic disequilibrium. Consequently, in the site of the clinic, preconscious fears and unconscious anxieties work together to disavow Black pain as hallucinatory or criminalize it as drug-seeking behavior. If, as Sadiya Hartman writes, “pain provides the common language of humanity; it extends humanity to the dispossessed and, in turn, remedies the indifference of the callous” (Hartman, 1997, p. 18), then pain as a language of dispossession, or loss, cannot be mobilized by a nonbeing who is already structurally possessed. Therefore, expressions, representations, performances, and enactments of pain necessary to set off the diagnostic cascade toward the recuperation of lost function, comfort, or health more broadly via treatment, or remedy, is unavailable if the violence that positions one structurally renders you not a degraded subject of loss but an abject object of absence.

Dismissal of Serena William’s chest pain post-emergency cesarian almost resulted in a fatal pulmonary embolism (How Serena Williams Saved Her Own Life, 2022). We can witness in sickle-cell treatment how the impossibility for representations of Black pain are only legible as drug-seeking behavior (Rouse, 2009).^12^ The inability to register Black suffering as pain can be further witnessed in the persistence of the false belief that Black people are less sensitive to pain than non-Black patients (Hoffman et al., 2016). Insofar as Black suffering is foreclosed to narrativization, clinical intervention, from en-casement to narrative medicine, that relies on a legible presentation of illness, or loss, through enactments of pain are all foreclosed. Enacted as a absence, there is no possibility for loss, hence nothing to recuperate let alone redeem. Only waking death—social, symbolic, material, and metaphysical.

Although the catastrophe Black emplotment poses for narrative proscribes en-casement, this does not translate into the absence of diagnoses—though it does often translate to the absence of anything resembling care. When it comes to Blackness, there is no shortness of diagnoses and pathologies—real, imagined, fantasized, or projected. The difference is these clinical judgements are made nondialogically, which is to say nonrelationally. As Foucault argues in *The Birth of the Clinic* (Foucault, 1963), the work of eighteenth-century French pathologist and anatomist Xavier Bichat integrated the penetrative gaze of the pathologist with the surface gaze of the clinician to enact the anatomo-clinical gaze.^13^ If the surface gaze of the clinician is dialogic and relies on enactments of pain and narratives of illness, then with regards to Black suffering we can postulate that only the former, penetrative gaze of the pathologist can be applied with any sort of consistency or reliability. Insofar as the attempts of narrative medicine fail to redress Black suffering because there was no legible narrative to be disciplined, distilled, or abstracted in the first place, the file for the Black “patient” is simply the product of the purely penetrative gaze in the abstract forms of pathology labs and imaging, surgical history and the like; it remains thoroughly nondialogic. Being nondialogic, diagnosis, real and imagined, relies solely on pathology and imaging, resulting in treatment that is far from patient-centered and has little concern for quality of life. Black suffering is, in effect, more than en-cased; it is always already entombed as a fact of Blackness. In other words, another process occurs when the entity that approaches the clinician does not, to be precise, rightfully possess a body nor have problems, but rather appears as a (non)being who, as W.E.B. Du Bois famously elaborates in *The Souls of Black Folks*, *is* a problem (Du Bois, 2014). When the illness narratives fail, the enactment of disease relies less on en-casement than it does entombment. Instead of being en-cased as patient-bodies, Black people are instead entombed by the nondialogic and penetrative gaze of the pathologist as diseased-flesh. Clinical apperception is so blunted with regard to Black pain such that disease is often only enacted when it is observed in an advanced stage. Consequently, anything resembling treatment in the wake of entombment often relies on more extreme and invasive forms of therapy. Treatment, let alone care, is nowhere in sight. For example, the persistent inability to manage let alone treat diabetes in Black patients often results in necrosis of the extremities and amputation (Lefebvre & Lavery, 2011).^14^ Black flesh cannot be treated allopathically, only discarded. When we consider the libidinal economy of anti-Blackness, we must entertain the disconcerting possibility that the disavowals of Black suffering, and the further pain and mutilation this often causes, is symptomatic of a desire for Black ruination. The persistence of the mutilation and immobilization of Black bodies after emancipation does not allow for such considerations, however disturbing, to remain uninterrogated. Black ruination and dereliction are not simply the inevitable effect of narrative foreclosure, but also an object of desire in and of itself, if only unconscious.

While narrative medicine grew out in response to the tendency in biomedical practice to condense and discipline illness narratives in ways the dehumanize people, rendering them patients, it is only effectively when applied to people—free, autonomous, self-possessed. When we consider the catastrophe Black social death poses for narrative at a metalevel in the context of the clinic it follows that narrative medicine as an intervention more than falls short when it comes to addressing Black suffering in the hegemonic paradigm of healing that is biomedicine. It fails. Narrative medicine can potentially rectify the reductionist experiment that transforms from a person in pain to a patient *with* problems; it cannot however redress suffering of a (non)being who *is* a problem (Du Bois, 2014). If the complex, non-linear illness narratives people deploy to represent pain and suffering are unsuitable for the anatomo-clinical gaze and clinical apperception, this general situation is exacerbated to the point of failure when the sufferer is Black. In general terms, for clinical apperception, suffering represented in illness narratives range from the illegible to the aporetic corresponding to the apparent alterity, or Blackness, of the speaker. When narrators are structurally positioned by relations of exploitation (i.e., women, immigrants, queer people, the indigenous, workers under capitalism, etc.) illness narratives, however cacophonous and chaotic, can enact disease and the patient-body, and thus initiate the treatment pathway. However, when speakers are structurally positioned by relations of domination, accumulation, and social death, illness narratives become illegible, aporetic, and unthinkable.

Insofar as Blackness is aporetic with respect to narrative writ large, the possibility for redemption from pain and the redress of suffering through narrative medicine is foreclosed to those fatally positioned by the constituent elements of social death (i.e., gratuitous violence, general dishonor, and natal alienation). Formal emancipation did not translate into the end of bondage (Hartman, 1997). Social death obliterates the capacity to narrativize suffering and catalyze the treatment pathway by making impossible the necessary transformation of the subjective experience of illness into an object of clinical understanding and allopathic intervention. Whether in sickness or in health, the emplotment of Black life is an impossibility in the wake of social death, rendering narrative medicine no less impotent with regard to Black suffering than clinical en-casement.^15^ The enactment of the Black patient-body is impossible precisely because there is no Black body to speak of, only flesh, which is always already pathological. It is precisely due to the fact that the presentation as diseased-flesh, not as a patient-body, that Black “subjects” are have been the preferred object of medical experimentation (Braun, 2014; Hornblum, 1998; Owens, 2017; Washington, 2008). Without a person to care for, but sharing an analogous anatomical structure, the Black “subject” is ideal object for excavating at the object of suffering-in-itself by any and all means necessary. As sentient and a priori diseased-flesh, the presentation of Blackness in the clinic is so great a stimulus that the clinician’s painstakingly cultivated gaze and clinical apperception are blunted and blinded by fear and pleasure simultaneously (2008). Instead of initiating a treatment pathway, the clinician is overwhelmed by unconscious fears, fantasies, and desires that render presentations of Black suffering as at once a terrifying specter of death (Warren, 2018) and a scene of perverse pleasure (Hartman, 1997). In either case, Black suffering is not an object of intervention, let alone care. Clinical apperception is blind to Black suffering, which is only rendered visible through raw anatomo-clinical gaze of the pathological anatomist which, occurring after death, is always already too late. Even then, it is overwhelmed by ubiquitous signs and markers of apparent deviance, rendering an accurate diagnosis, or in this case cause of death, impossible. Even when clearly murdered and brutalized by the state, autopsies of Black victims of police brutality remain ambiguous and suspect. The alleged suicide of Sandra Bland or the death of Freddie Grey, both in police custody, are but two examples.^16^

Insofar as paradigmatic Black social death obliterates the possibility for a prior plenitude, the enactment of a patient-body is foreclosed; all that is available for clinical apperception is a priori diseased-flesh that lacks the capacity for redemption, recuperation, or restoration. The foreclosure of Black subjectivity renders biomedical paradigms of healing, including narrative medicine, impotent with regards to redressing Black suffering. To quote Wilderson again,

[w]hen violence is the law, and not the effect of its enforcement, it presents the rules of narrative with a crisis; because what we have is a situation that resists retelling, for the simple reason that narrative’s causal principle, the ghost in the machine we call the causal logic (or ‘because principle’) of the story, is missing. (2020, pp. 89–90)

As the fantastical embodiment of *pathos* itself, the only redress to Black suffering in the secular-republican tradition comes through death. If the secular-republican tradition and the biomedical paradigm of healing therein is at a loss with regards to the redress of suffering civil society’s citizens and subjects, it is a complete and total failure when it comes to secular man’s phantasmagoric and constitutive other: the so-called Negro, or the Black.^17^ There is a never a space or time when the Black could be considered well such that suffering, acute or chronic, becomes legible to narrativization. Even if presented as such, there is a fundamental doubt that the narrative is or even could be true. Thus, robbed of a body and the capacity for representation, let alone recognition, within hegemonic structures of clinical understanding and reasoning, the violent enactment of paradigmatic social death renders the socially dead too Black for care (2020, p. 3). Insofar as the Black “body” remains the essential stimulus to anxiety, a phobogenic object, and a site of absolute dereliction, of absence, biomedicine can only approach Black suffering as the effect of naturally diseased-flesh, and as such never therapeutically, but always experimentally, violently, and desirously.

In summary, the illegibility and moreover unthinkability of Black life or health due to social death and domination renders interventions within the biomedical paradigm of healing impotent in the face of Black suffering. Moreover, the incapacity to narrativize Black suffering makes the insertion of Black subjectivity an exercise in futility. One cannot rehumanize that which was never properly Human—which is to say free, healthy, and socially alive. Thus, when approached from the position of Blackness, the interrogation of the biomedical paradigm of healing elevates the critique of narrative medicine to consider how the problem is bigger than rendering others’ embodied experience to suffering legible to clinical apperception. It throws into relief how the structure of anti-Black violence renders the embodied suffering of Black people illegible within biomedical paradigms of healing. Allopathic healing becomes is an impossibility, fundamentally rooted in the fact that for Black (non)being, there is no prior moment of plenitude, health or the absence of suffering. Narrative medicine can ameliorate loss, but never absence.

That being said, just as the patient-body of the Human is an enactment, so too is the allegedly a priori diseased-flesh of the Black “patient.” The enactment of paradigmatic Black social death is the structural assemblage of coordinated discourse, practices, and institutions that constitute the quotidian, or the ordinary, the everyday, and the mundane of civil society more broadly (Hartman, 1997). Therefore, in recognizing that the subject-object status of the Black is not simply performed or constructed but rather the product of a coordinated enactment it becomes clear that, far from divine decree, Black social death can and must be destroyed (Wilderson III, 2020, p. 103).

## Abolition Medicine: A Politics of the Imperative, an Ethics of the Real

The ever-expansive inclusionary gesture must thus be displaced by another more radical approach: *an ethics of the real, a politics of the imperative, engaged in its interminably downward movement*. This daunting task entails making necessity out of virtue, as it were, willing the *need* for the black radical imagination and not just its revisable demand.

–Jared Sexton, Afro-Pessimism: The Unclear Word

As I have argued, the biomedical style of reasoning that governs hegemonic paradigms of healing within the secular-republican tradition cultivates a faculty of clinical apperception that reduces and abstracts from illness narratives disease as an object of suffering-in-itself. Suffering is thus approached as an object of knowledge through abstraction, rather than an existential condition part and parcel with being alive irreducible to objectification. In the wake of secular finitude, suffering is understood as an oppressive limit to the realization of freedom (Asad, 2003), marginalized from the triumphant narrative of the human and approached through relations of opposition, or enmity. As an allopathic (i.e., *allo*- opposite, *pathos*- suffering) paradigm of healing, biomedicine is oriented against suffering and redresses it through its opposite. Sulfonylureas to stimulate the pancreatic production of insulin for the uptake of glucose becomes the preferred treatment for diabetes to compensate for blood-sugar values beyond a certain range, rather than changes in diet or exercise. Conversely, exercise becomes the remedy for a sedentary life in late capitalism and prevention of associated disease-states, crowding out a fundamental restructuring of political economy as a form of care. However, care within the secular-republican order of things forgoes structural intervention for a disavowal of pain and valorization of pleasure to achieve a state of uninterrupted enjoyment wherein freedom can be fully lived and humanity realized (Sexton, 2015). Slavoj Žižek’s famous example of the chocolate laxative comes to mind (Žižek, 2006), whereby one can indulge in that which causes constipation (i.e., chocolate) in the form of a laxative. Insofar as Blackness is a positioned as “site of absolute dereliction at the level of the Symbolic,” made coterminous with unfreedom and suffering through the gratuitous violence, natal alienation, and dishonor of social death, then to say the biomedical paradigm of healing is opposed to suffering is to say it is opposed to Blackness as it is symbolically, materially, and metaphysically enacted. The incapacity for biomedicine to move with, rather than against, suffering renders it incapable of moving with and not against Blackness. In short, biomedicine is against suffering, against Blackness, in ways that render it incapable of promoting Black life. Clinical apperception conceives of suffering as the condition of Black life that can only be resolved through death.

Despite what has been said, one might still be tempted to argue that the biomedical approach of stripping away the narrative, empirical, subjective and sensational aspects of illness to get at the object of suffering-in-itself is not necessarily problematic. After all, one could argue that biomedicine has demonstrated a marked ability to alleviate symptoms, treat disease, and prolong biological life. However, the alleviation of symptoms, treatment of disease, and extension of biological life does not necessarily translate to the redress of suffering, which is social, lived, and embodied. Arguably, it is precisely the impact of illness and suffering on lived, embodied experience, rather than deviation from an allegedly universal standard or norm, that drives one to seek care in the first place. Furthermore, we cannot take for granted the categories of symptom and disease, categories which are themselves unique to a particular onto-epistemic tradition and paradigm of healing. In other words, particular sensations are understood as signs of illness precisely because they misalign with a no less particular understanding of health, which varies both from tradition to tradition and across space and time.

When we consider how the biomedical enactment of disease and the patient-body operates through the always violent overdetermination of what it means to suffer from the perspective of Enlightenment thought, it becomes all the more apparent how the clinical gaze and apperception is exercise of power that crowds out other ways of redressing suffering. Whereas one could perhaps argue that the physical sensations which are intuited as suffering are more or less shared among human beings—bullets wound uniformly—one would have much more difficulty arguing that the synthesis of these sensations experienced as suffering under the categories of symptoms, illness, and disease are universal. Even if we are to assume for a moment that across all onto-epistemic traditions and paradigms of healing, the spatiality of the human body and the temporality of pain that provide the conditions of possibility such that the sensational experience of suffering is intuited in the same way—a huge assumption, to say the least—nevertheless the subsequent conceptualization and redress of these depends on styles of reasoning derivative from a particular discursive and embodied tradition, for which there are many. Even if the physical experience of pain is universal, its meaning across space and time is far from it. Given that the apprehension of experiences intuited as suffering into understandings of illness are mediated by a discursive tradition, or styles of reasoning, we cannot assume that everyone apprehends, cognizes, let alone rationalizes the sensation of pain in the same way. Furthermore, the distinction between pleasure and pain is not unambiguous, itself being socially mediated (Asad, 2011).

Given the failure of current clinical models and frameworks, from cultural humility and structural competency to narrative suffering, to redress Black suffering, a potentially radically new space of theory and praxis has emerged called abolition medicine. In its commitment to a Black radical imagination that exceeds demands for reform, abolition medicine poses a fundamental challenge to hegemonic understandings of the body, as anatomical, and paradigms of healing, as biomedical. When taken to its logical conclusion, an abolition framework demands not simply the reform of medicine but its fundamental restructuring, calling into question clinical apperception and biomedical modes of understanding health, illness, and life itself. Abolition does not translate into a call for the reformation of biomedicine, but a demand for the reimagination and reinvention of what it means to care, to live and to die in the wake of catastrophe and afterlife of slavery. In order to invent a new way of caring into existence, what is required is to sit with the problem of Black suffering. As Wilderson argues, it requires us to make a “home in the hold of the ship and burn it from the inside out” (2020, p. 323).

If as Turner and Wallace write,

[t]he danger of abolition being taken up quickly – even if fervently – is just this: that, decontextualized from its historic Black and Indigenous foundations, abolition can become nothing more than a pretty and ephemeral metaphor (Tuck and Yang, 2012) stripped of its inherent nuances, ancestral learnings and ultimately its power. (Turner et al., 2023)

then to be liberatory, abolition medicine must take seriously different paradigms of body, traditions of health and illness, and understandings of life and death. It is not my intention to point toward any particular discursive and embodied tradition or paradigm of healing as a way out, only to emphasize the particular limits and failures of the secular-republic tradition and the biomedical paradigm of healing with regard to the redress of Black suffering. In any case, an abolitionist approach that remains committed to a biological conception of life, the anatomical body, and secular-republican civil society wherein desubjectivation through processes ranging from the partial to the absolute, from en-casement to entombment, is the first step in the pathway to care will never redress Black suffering. Prior investments in biological understandings of life crowd out any potential of an abolitionist approach to medicine, boxing in a Black radical imagination into conceptual frameworks that were built through anti-Blackness itself. Drawing on the work of Hortense Spillers, the remedy requires approaching the question of suffering from a position vestibular to history and culture, from an antechamber to an anti-Black world (Spillers, 2003), a conceptual before that Jared Sexton describes as ante-anti-Blackness (Sexton, 2012), which walks the line between optimism and pessimism, between life and death.

However, as Wilderson cautions,

If we are to be honest with ourselves, we must admit that the “Negro” has been inviting whites, as well as civil society’s junior partners, to the dance of social death for hundreds of years, but few have wanted to learn the steps. They have been, and remain today—even in the most anti-racist movements, like the prison abolition movement—invested elsewhere. This is not to say all oppositional political desire today is pro-white, but it is usually anti-Black, meaning it will not dance with death. (2003)

To the extent an abolitionist approach to medicine, while ostensibly far from being pro-White, is invested in biological conceptions of life and death, it will fail to do what is required for the redress of Black suffering, which is to say dance with death. A truly abolitionist approach to medicine cannot be applied without destroying the anti-Black conceptualization of life itself, without dancing with death. After all, high life-expectancies in unexpected places like Cuba where the health expenditures per capita and daily cost of living are remarkably low compared to the US^18^ cannot be disentangled from their political commitments and revolutionary praxis. From this perspective, insurrection becomes a form of care. The general refusal to dance with death born in the wake of secular finitude where you only live once is an impediment to “willing the need for a Black radical imagination” (Sexton, 2016, p. 6) If abolition is tantamount to the dance with death, then it can only ambivalently engage with the hegemonic discourses and practices of biological life. Identifying and elaborating the points of refusal and reconciliation, stealing as it were the value in biomedicine and leaving the rest, is the labor of a Black radical imagination just as the redress of Black suffering is its invention. Arguably, this is best pursued by those who already have an ambivalent attachment to life writ large, for whom death is only the beginning (Cherian, 2023). Through such a loose and ambivalent attachment to life and the world, perhaps rather than opposing life with death, illness with health, subject with object, those who dance with death might approach the condition of suffering as foremost among the vicissitudes of existence, neither disavowing or valorizing it but rather struggling through and with it.

Inspired by the recognition that Black suffering is always already too opaque and aporetic for secular-republican forms of care, we must refuse the desire to violently know the incalculable vicissitudes of life as an object-in-itself. In its place, demand neither revision nor reform, but rather pursue “an ethics of the real, a politics of the imperative, engaged in its interminably downward movement…making necessity out of virtue” (Sexton, 2016, p. 6) In the words of psychiatrist and revolutionary Frantz Fanon, “what matters is not to know the world, but to change it.”

## Footnotes

1 The concept of ante-anti-Blackness speaks to an unthinkable “before” (i.e., *ante*-before) a world structured by anti-Blackness. Rather than a temporal before, it draws from the concept of “vestibularity” developed by Hortense Spillers in *Mama’s Baby, Papa’s Maybe.* It asserts that an ante-anti-Black world evades thought let alone signification in an anti-Black world and must be invented. Insofar as the act of Black invention is simultaneously one of destruction, it could be termed abolition.

2 Drawing on the work of Talal Asad, I am thinking of secular-republicanism as one emerging out of Western Europe from the Kantian tradition wherein secular disenchantment by way of empiricist science converges and evolves alongside discourses of personal liberty, freedom and equality. In the formation of Western European and North American nation-states, religious “freedom” is secured through sequestering it to the private domain, leaving the secular public to be governed by Reason. However, as Asad and other anthropologists of secularism have pointed out, such neat separations are complicated by how the religious inflects the secular and vice versa. Nevertheless, the point of the hyphenated term is to emphasize how secularism and liberalism converge in ways that are important not only for politics, but epistemology and ontology more broadly. See (Asad, 2003, 2018).

3 All the paperwork about any single patient comes together in a file. Summaries turn a specific patient’s atherosclerosis into a single object (Mol, 2003, p. 56).

4 For example, in the case of atherosclerosis which is the focus of Mol’s ethnography, or what she calls praxiography, the object of atherosclerosis emerges through the coordination of clinical and pathological enactments of atherosclerosis, wherein the former atherosclerosis emerges through dialogic, tactile and embodied processes of the interview and physical exam in the clinic and in the latter it emerges through scopic, penetrative and disembodied processes of dissection and microscopy in the pathology lab.

5 After the reductionist experiment of dissecting the patient’s narrative into individual problems, these pieces can then be reconfigured to produce a coherent narrative….the problem-oriented patient presentation effectively en-cases the human patient for efficient biomedical consumption (Holmes and Ponte, 2011, p. 179).

6 “objects are therefore given to us by means of sensibility, and it alone affords us intuitions; but they are thought through the understanding, and from it arise concepts… [therefore] all thought…must ultimately be related to intuitions, and thus, in our case, to sensibility, since there is no other way in which objects can be given to us” *Critique of Pure Reason*, p. B672.

7 “the supreme principle of all intuition in relation to the understanding is that all the manifold of intuition stand under condition of the original synthetic unity of apperception” (Kant, 1999, p. B136).

8 “we are acquainted with nothing except our way of perceiving them [i.e., appearances], which is peculiar to us, and which therefore does not necessarily pertain to every being, though to be sure it pertains to every human being. We are concerned solely with this” (Kant, 1999, p. B60).

9 As Kant says “judgment is nothing other than the way to bring given cognitions to the objective unity of apperception. That is the aim of the copula [between sensibility and understanding] is to distinguish the objective unity of given representations from the subjective” (Kant, 1999, p. B142).

10 “Blackness is the site of absolute dereliction at the level of the Symbolic, for Blackness in America generates no categories for the chromosome of history, and no data for the categories of immigration or sovereignty. It is an experience without analog—a past without a heritage” (Wilderson III, 2003, p. 16).

11 As Wilderson writes, “the narrative arc of the slave who is Black…is not an arc at all, but a flat line, what Hortense Spillers calls ‘historical stillness’: a flat line that moves from disequilibrium, to a moment in the narrative of faux-equilibrium, to disequilibrium restored and/or rearticulated…. Blackness is social death, which is to say that there was never a prior moment of plenitude, never a moment of equilibrium, never a moment of social life….To put it differently, the violence that both elaborates and saturates Black ‘life’ is totalizing, so much so as to make narrative inaccessible to Blacks” (Wilderson III, 2020, pp. 102, 226).

12 Carolyn Rouse’s ethnography of sickle-cell disease demonstrates how Black “patients”—even children—are accused of drug-seeking when seeking medication to alleviate systemic pain due to systemic flareups.

13 Foucault describes the emergence of the anatomo-clinical gaze as the synthesis of the surface gaze and the anatomical gaze, or the union of the clinic and the pathology lab, within European medical practice in the nineteenth century.

14 Amputation rates among Black people are 70% greater than among Whites and 114% greater than non-Hispanic Whites.

15 “Between me and the other world there is ever an unasked question: unasked by some through feelings of delicacy; by others through the difficulty of rightly framing it. All, nevertheless, flutter round it. They approach me in a half-hesitant sort of way, eye me curiously or compassionately, and then, instead of saying directly, How does it feel to be a problem?” Dubois, p. 1.

16 The autopsy of Sandra Bland reported suicide by asphyxiation as cause of death. Freddie Grey reported “high-energy injury” as the COD.

17 Charle’s Markman’s decision to translate Fanon’s *Le Negre* as *The Black* is due to the liminal subject-object status of Black positionality. For our purposes, it is thus not precise to say the Black subject, or the Black person, or the Black patient without ““ (i.e., the Black “subject”). Within critical Black studies the use of the term “the Black” is more appropriate to emphasis the limits of grammar with regard to narrativize Black experience in the wake of social death. Neither a full subject nor an absolute object, the so-called Negro, or the Black, is a speaking implement.

18 By current estimates using WHO statistics, in Cuba, per Capita health spending is $1336; in the US it is $13493, over 10×.
